# Hepatocystin is Essential for TRPM7 Function During Early Embryogenesis

**DOI:** 10.1038/srep18395

**Published:** 2015-12-16

**Authors:** Jeffrey D. Overton, Yuko Komiya, Courtney Mezzacappa, Kaushik Nama, Na Cai, Liping Lou, Sorin V. Fedeles, Raymond Habas, Loren W. Runnels

**Affiliations:** 1 Rutgers-Robert Wood Johnson Medical School, Dept. of Pharmacology, Piscataway, 08854, USA; 2 Temple University, Dept. of Biology, Philadelphia, 19122, USA; 3 Yale University School of Medicine, Dept. of Internal Medicine, New Haven, 06510, USA

## Abstract

Mutations in *protein kinase C substrate 80K-H (PRKCSH)*, which encodes for an 80 KDa protein named hepatocystin (80K-H, *PRKCSH*), gives rise to polycystic liver disease (PCLD). Hepatocystin functions as the noncatalytic beta subunit of Glucosidase II, an endoplasmic reticulum (ER)-resident enzyme involved in processing and quality control of newly synthesized glycoproteins. Patients harboring heterozygous germline mutations in *PRKCSH* are thought to develop renal cysts as a result of somatic loss of the second allele, which subsequently interferes with expression of the TRP channel polycystin-2 (PKD2). Deletion of both alleles of *PRKCSH* in mice results in embryonic lethality before embryonic day E11.5. Here, we investigated the function of hepatocystin during *Xenopus laevis* embryogenesis and identified hepatocystin as a binding partner of the TRPM7 ion channel, whose function is required for vertebrate gastrulation. We find that TRPM7 functions synergistically with hepatocystin. Although other N-glycosylated proteins are critical to early development, overexpression of TRPM7 in *Xenopus laevis* embryos was sufficient to fully rescue the gastrulation defect caused by loss of hepatocystin. We observed that depletion of hepatocystin in *Xenopus* laevis embryos decreased TRPM7 expression, indicating that the early embryonic lethality caused by loss of hepatocystin is mainly due to impairment of TRPM7 protein expression.

In this study we investigated the role of hepatocystin during embryogenesis and its functional relationship to the TRPM7 ion channel, which our studies found to biochemically and functionally interact with hepatocystin. TRPM7 is a divalent-selective channel permeable to Mg^2+^, Ca^2+^, as well as trace metal ions such as Zn^2^+ 1,2. The channel is bifunctional, containing an alpha kinase domain at its COOH-terminus^3^. Global deletion of *TRPM7* from mice results in early embryonic lethality by E7.5^4^. In *Xenopus laevis*, TRPM7′s channel, but not its kinase, was found to be critical to the regulation of gastrulation and neural fold closure during *Xenopus laevis* embryogenesis^5^.

In the ER, hepatocystin functions as the noncatalytic beta subunit of Glucosidase II, which catalyzes glucose trimming of newly synthesized glycoproteins and is involved in ER protein quality control^6^,^7^,^8^. Glucosidase II cleaves the first glucose prior to entry of a glycoprotein into the calnexin/calreticulin cycle^9^,^10^,^11^,^12^. If a glycoprotein is properly folded, Glucosidase II cleaves off a second glucose residue to allow for transition of the glycoprotein out of the ER to the Golgi apparatus. If however the glycoprotein is improperly folded, it is ultimately targeted for degradation by the ER-associated degradation (ERAD) pathway^9^,^12^. Hepatocystin contains an NH_2_-terminal signal sequence for translocation across the ER membrane, a low-density lipoprotein class A domain, two Ca^2+^-binding EF hands, a glutamic acid-rich segment, a region with low homology to the mannose 6-phosphate receptor (MRH), and a COOH-terminal His-Asp-Glu-Leu (HDEL) ER retention signal (Fig. 1A)^7^. Hepatocystin’s ER luminal retention signal is required for the function of the glucosidase II holoenzyme^13^ Genetic loss of hepatocystin causes autosomal dominant polycystic liver disease (ADPLD), which is characterized by bile duct cyst formation throughout the liver parenchyma^14^,^15^. It is hypothesized that liver cyst development is driven by somatic second hit mutations that affect wild type alleles of biliary type cells during early hepatic organogenesis^16^. Loss of hepatocystin impairs glucosidase II-dependent glucose trimming of critical N-glycoslyated proteins, such as the TRP channel PKD2, which have been shown to function in a genetic interaction network with *PRKCSH*^17^,^18^. Here we report an analogous role for hepatocystin in supporting the expression and function of the TRPM7 ion channel during early embryonic development.

## Results

### Hepatocystin is a TRPM7 binding protein

**Figure 1:**
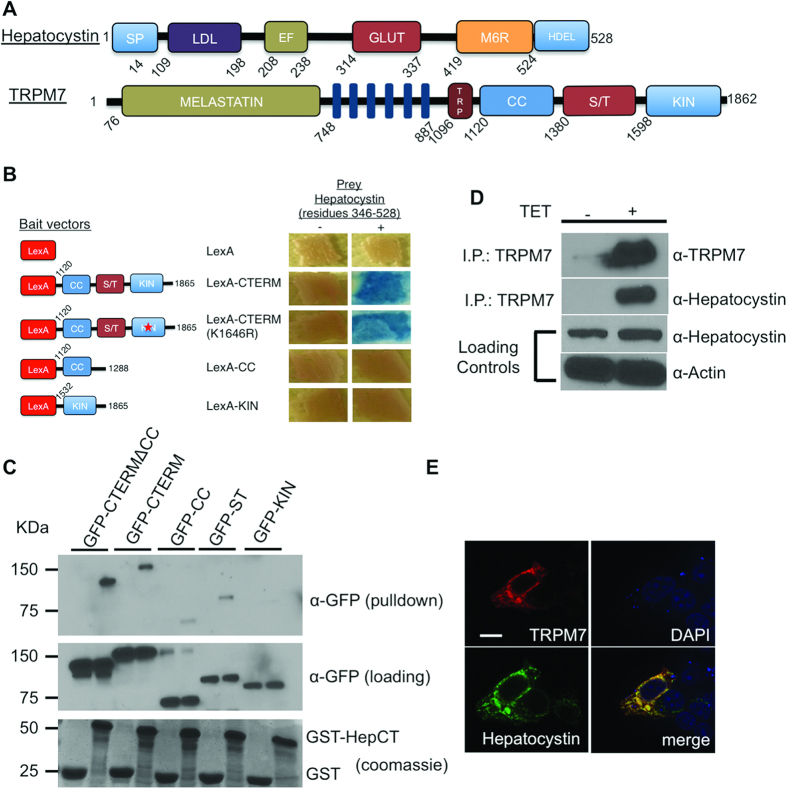
Hepatocystin interacts with TRPM7. (**A**) Schematics of the linear structures and domains of TRPM7 and hepatocystin. TRPM7 contains an NH_2_-terminus melastatin domain, six transmembrane domain, TRP box, as well as coiled-coil (CC), serine/threonine rich domain (ST), and functional alpha kinase (KIN) domains. Hepatocystin contains a leader signal peptide (SP), a low-density lipoprotein class A (LDL) domain, two Ca^2+^-binding EF hands, a glutamic acid-rich segment (GLUT), a region with low homology to the mannose 6-phosphate receptor (MRH), and a COOH-terminal His-Asp-Glu-Leu (HDEL) ER retention signal. (**B**) Directed yeast-two-hybrid assay using the indicated fragments of TRPM7 COOH-terminus fused to LexA as bait and Gal4 fused residues 346-528 of hepatocystin as prey. LexA fused to Laminin (Lex-Lam) served as a negative control. (**C**) Pulldown purification assay using GST fused to residues 346-528 of hepatocystin (GST-HepCT) against HEK-293 cell lysates containing the indicated mfGFP-tagged TRPM7 COOH-terminal fragments. (**D**) Endogenous hepatocystin co-immunoprecipitates with HA-tagged TRPM7 heterologously expressed in HEK-293T cells. (**E**) Immunocytochemistry of HEK-293T cells transfected with HA-tagged TRPM7 and FLAG-tagged hepatocystin (Green) show that the two proteins co-localize in the endoplasmic reticulum (ER). A scale bar of 20 microns in length is indicated.

To identify potential regulatory binding partners of TRPM7 we conducted a yeast two hybrid (Y2H) screen of a rat brain library using the COOH-terminus of TRPM7, which contains a coiled-coil domain, serine/threonine rich domain and functional alpha kinase domain (Fig. 1A). The screen identified a number of potential interacting partners (Supplementary Table I), including a COOH-terminal fragment of hepatocystin (residues 346–528) containing the protein’s MRH and HDEL motifs. We then used a directed Y2H assay to confirm the interaction between TRPM7′s COOH-terminus and hepatocystin and showed that TRPM7′s coiled-coil and alpha-kinase domains do not interact with hepatocystin (Fig. 1B). This assay also revealed that a kinase-inactive mutant (K1646R) of TRPM7 is also capable of interacting with hepatocystin, indicating that a functional kinase domain is not required for the protein-protein interaction. We next used a pull-down purification assay using GST fused to residues 346–528 of hepatocystin (GST-hepatocystin) to further delineate the region within TRPM7 COOH-terminus that interacts with hepatocystin. GST-hepatocystin, but not the GST negative control, interacted with the full length TRPM7 COOH-terminus (GFP-CTERM) and the COOH-terminus lacking the coiled-coil (GFP-CTERMΔCC), but interacted more weakly with the isolated coiled-coil domain (GFP-CC) (Fig. 1C) and serine-threonine-rich domains (GFP-ST). Similar to what was observed in the directed Y2H assay, GST-hepatocystin did not interact with the isolated kinase TRPM7 domain (GFP-KIN). These results indicate that hepatocystin interacts with TRPM7′s ST domain. As the ST-domain has been implicated in kinase substrate recognition by TRPM7, we investigated whether TRPM7 phosphorylates hepatocystin^19^. However, an *in vitro* kinase assay demonstrated that compared to a commonly used substrate for protein kinases, myelin basic protein (MBP), hepatocystin is not a good substrate for TRPM7′s kinase (Supplementary Fig. 1). We next determined whether the full-length proteins interacted *in vivo* and found that endogenous hepatocystin co-immunoprecipitated with TRPM7 heterologously expressed in HEK-293 cells (Fig. 1D). Since hepatocystin contains an ER-retention signal and has been shown to be primarily expressed in the ER, we then investigated whether TRPM7 co-localized with hepatocystin to the ER^7^. Immunocytochemistry analysis of transiently transfected HA-tagged TRPM7 expressed in HEK-293 cells and FLAG-tagged hepatocystin revealed co-localization of both proteins to the ER (Fig. 1E). Collectively, these results indicate that TRPM7 likely encounters and interacts with hepatocystin in the ER. Previous studies have demonstrated that homozygous deletion of either gene from mice is embryonically lethal^4^,^17^. As our prior investigation of the role of TRPM7 during early development uncovered a role for TRPM7 channel activity in gastrulation cell movements and neural fold closure, this motivated us to more closely investigate the function of hepatocystin during embryogenesis and to determine whether the function of the two proteins functionally intersect during early development^5^.

### Hepatocystin is required for gastrulation during *Xenopus laevis* embryogenesis

**Figure 2:**
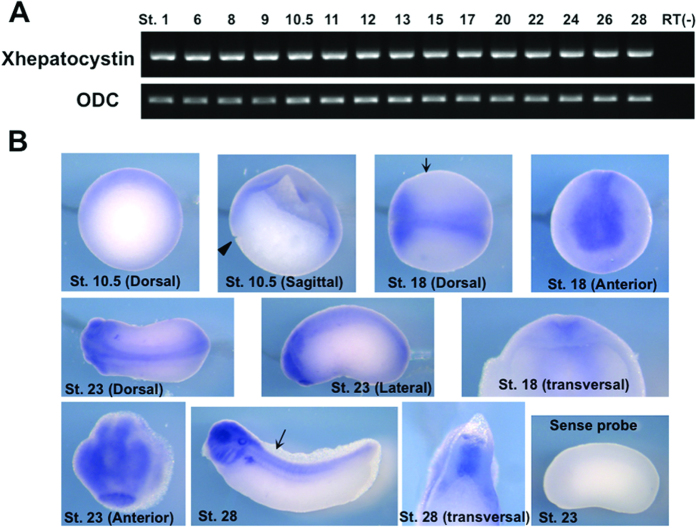
Expression pattern of Xhepatocystin during early development. (**A**) Xhepatocystin is expressed throughout embryogenesis as monitored by RT-PCR analysis. Ornithine decarboxylase (ODC) was used as a loading control. (**B**) Expression pattern of Xhepatocystin at selected developmental stages as analyzed by whole-mount *in situ* hybridization using a Xhepatocystin anti-sense probe. A sense probe was used as a negative control. Arrows indicate the region where the embryo was vertically sectioned and examined. ST: stage. Xhepatocystin was expressed in dorsal ectoderm and dorsal mesoderm. In neurula stage embryos (stage 18 & 23) Xhepatocystin was highly expressed in the neural plate and anterior neural fold. At the early tadpole stage, Xhepatocystin was found expressed in the head, neural tube and notochord. Later in development, strong expression was observed in head, eyes, spinal cord, notochord, liver and kidney.

Deletion of *PRKCSH* in mice results in embryonic lethality before embryonic day E11.5, however, the reason for this has not been determined^17^. To investigate the role of hepatocystin during early embryogenesis, we first cloned *Xenopus laevis* hepatocystin (Xhepatocystin), a protein that shares a sequence identity of 61% with the human protein. Xhepatocystin domain architecture (Fig. 1A) is similar to that of its vertebrate orthologs, exhibiting high sequence identity and similarity within its 6 structural domains or motifs. We next examined the temporal and spatial expression patterns of Xhepatocystin mRNA during *Xenopus* development. RT-PCR analysis demonstrated ubiquitous expression of Xhepatocystin maternally to the tadpole stage (Fig. 2A). *In situ* hybridization of gastrula stage embryos (stage 10.5) showed expression of Xhepatocystin in dorsal ectoderm and dorsal mesoderm (Fig. 2B). In neurula stage embryos (stage 18 & 23) Xhepatocystin was highly expressed in the neural plate and anterior neural fold. At the early tadpole stage, Xhepatocystin was expressed in the head, neural tube and notochord. Later in development, strong expression was observed in head, eyes, spinal cord, notochord, liver and kidney. In our previous study, we also observed ubiquitous expression of *Xenopus laevis* TRPM7 (XTRPM7) maternally to the tadpole stage by RT-PCR and *in situ* hybridization showed that XTRPM7 is similarly expressed in the neural plate at the neurula stage and in kidney at tadpole stage^5^. The overlapping expression patterns of Xhepatocystin and XTRPM7 indicated the potential for these two proteins to functionally interact *in vivo*.

**Figure 3:**
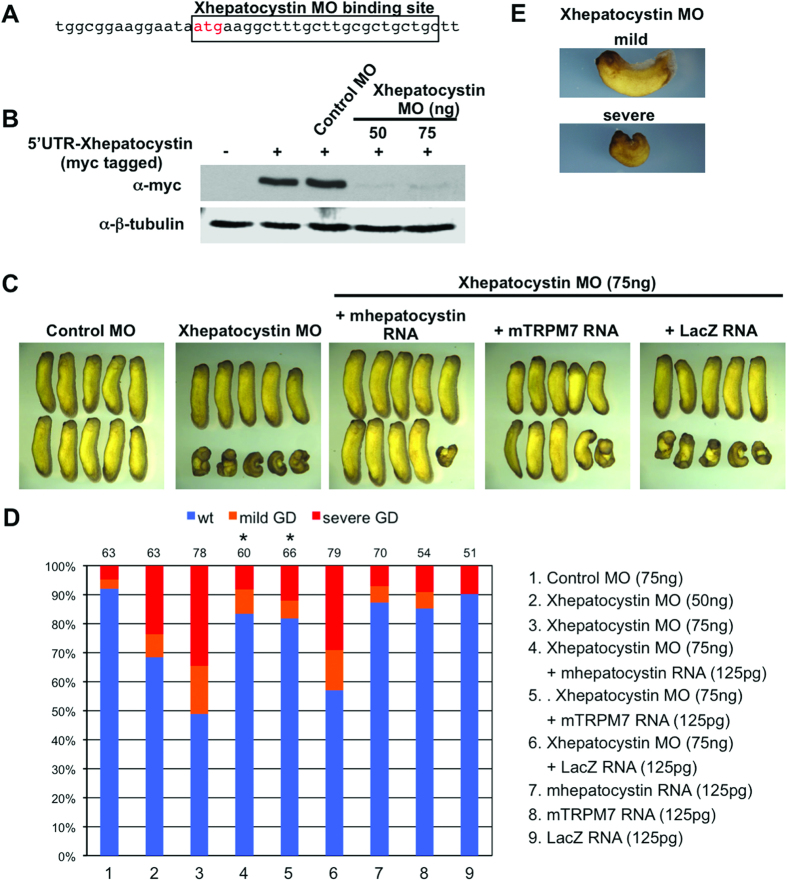
Xhepatocystin is required for gastrulation. (**A**) Schematic diagram of Xhepatocystin morpholino (MO) binding sites upstream and overlapping the start site of Xhepatocystin. (**B**) Western blot analysis shows that injection of the Xhepatocystin MO (50 and 75 ng), but not the control MO (75 ng), can effectively inhibit translation of Myc-Xhepatocystin 5′ UTR injected RNA (250 pg). β-tubulin is shown as a loading control. (**C**) Injection of Xhepatocystin MO into the dorsal blastomeres of the 4-cell stage embryos inhibited gastrulation. The gastrulation defect phenotype was rescued by co-injection of mouse hepatocystin RNA and mouse TRPM7 RNA (125 pg), but not LacZ RNA (125 pg), with the Xhepatocystin MO (75 ng). Injection of the control MO (75 ng) produced no phenotype. (**D**) Quantification of phenotypic results from (**C**). Phenotypes were scored according to the severity of the gastrulation defect (GD) at the tadpole stage (**E**). The injections were repeated at least three times. The ability of co-injection of mouse hepatocystin and mouse TRPM7 RNA with Xhepatocystin MO to rescue the gastrulation phenotype caused by the Xhepatocystin MO was statistically significant (*P<0.05). The collective total number of injected embryos from all experiments is indicated above each bar.

In our earlier study, we reported a requirement for TRPM7 in controlling gastrulation cell movements and neural fold closure during *Xenopus laevis* embryogenesis^5^,^20^. We found that during gastrulation TRPM7 specifically regulates cell polarity and migration during convergent extension movements by controlling the activation of Rac during non-canonical Wnt signalling. We next sought to determine whether Xhepatocystin possesses a similar regulatory role. To accomplish this we depleted Xhepatocystin protein by injection of anti-sense morpholino oligonucleotide into embryos at the 4-cell stage (Xhepatocystin MO; Fig. 3A) and observed its effect during development. The Xhepatocystin MO effectively inhibited translation of a Myc-tagged Xhepatocystin construct when injected into *Xenopus* embryos (Fig. 3B). Injection of Xhepatocystin MO into the dorsal marginal zones of the four-cell embryo dose-dependently impaired axis extension and interfered with neural tube closure, while injection of a similar dose of a control MO had no significant effect (Fig. 3C&D). The phenotype produced by dorsal injection of the Xhepatocystin MO was divided into two classes: severe and mild (Fig. 3E). In the severe class, axial extension was impaired, resulting in an extreme dorsal-flexure in the embryos, and the neural tube failed to close. In addition, anterior structures such as the head and eyes were reduced in size. In the mild class, there was delay in neural tube closure and axis extension was mildly impaired, which resulted in short and dorsally curved embryos. The penetrance of the Xhepatostatin MO was relatively mild (approximately 50 percent), which was likely due to the high maternal expression of Xhepatocystin (Fig. 2A). Nevertheless, Xhepatocystin MO induced gastrulation defects that could be rescued by co-injection of mouse hepatocystin RNA, which does not contain the MO binding site (Fig. 3D). Importantly, co-injection of LacZ RNA did not rescue the Xhepatocystin-induced gastrulation defects, indicating the specificity of Xhepatocystin MO. Dosages of the Xhepatocystin MO of 100 ng and above produced non-specific toxic effects, which could not be rescued by the hepatocystin RNA. Collectively, these results demonstrate a critical and specific role for Xhepatocystin for gastrulation and neural fold closure.

**Figure 4:**
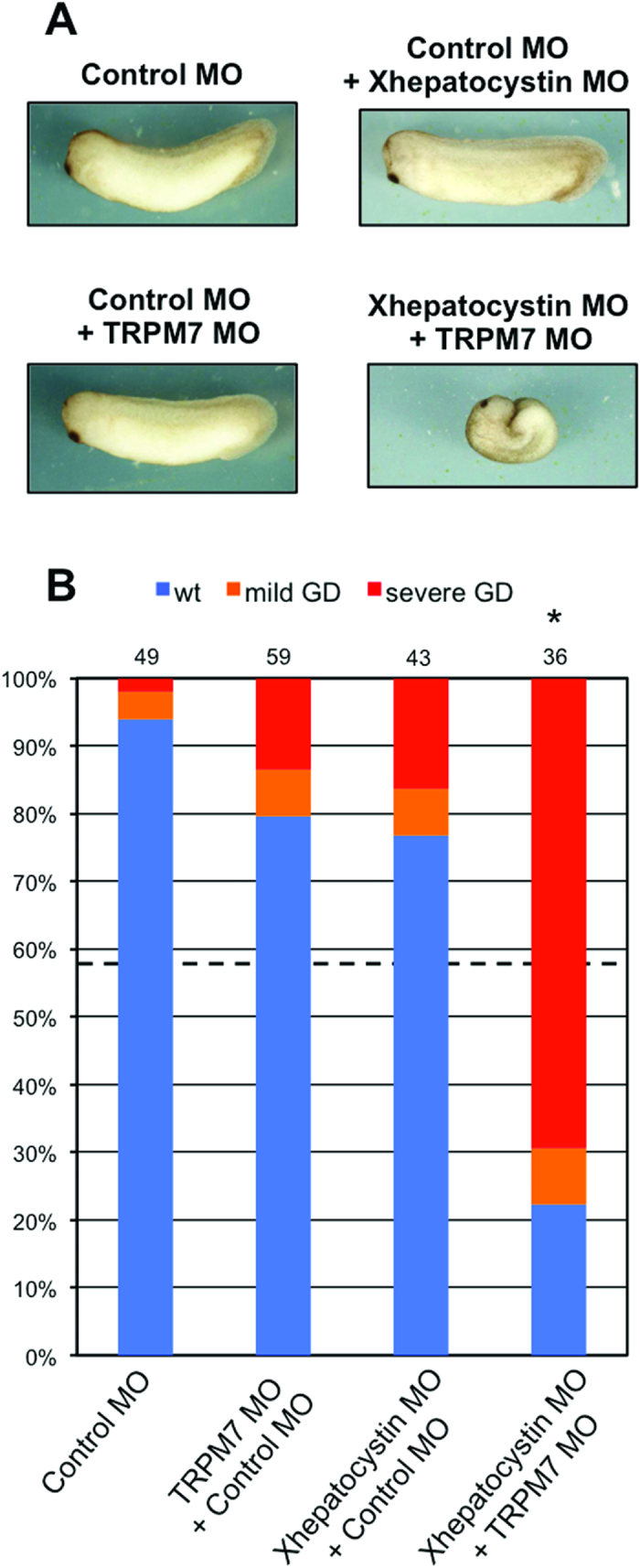
TRPM7 and hepatocystin functionally interact. (**A,B**) Co-injection of Xhepatocystin MO (15 ng) and XTRPM7 MO (15 ng) synergistically inhibit gastrulation, but have little or no effect when injected separately. (**B**) Phenotypes were scored according to the severity of the gastrulation defect (GD) at the tadpole stage. The dotted line in (**B**) indicates the amount of gastrulation defects that would be expected if the effects were additive (43%). The injections were repeated three times. Statistic analysis indicated that the number of embryos exhibiting gastrulation defects (77%) by co-injection of Xhepatocystin and XTRPM7 MOs was significantly different (P=0.05) from what would be expected if the effects were additive (43%). The collective total number of injected embryos from all three experiments is indicated above each bar.

Given the similarity between the spatial and temporal expression pattern of Xhepatocystin and XTRPM7 and the gastrulation defects phenotypes produced by depletion of either protein, we next asked whether two proteins functionally interact. To address this question, embryos were injected with sub-threshold levels of the Xhepatocystin MO or XTRPM7 MOs. Injection of fifteen nanograms of Xhepatocystin MO or of TRPM7 MO with 15 ng Control MO caused mild gastrulation defects in 10–20 percent of the embryos. However, when Xhepatocystin MO and XTRPM7 MOs were co-injected, the number of defected embryos increased to 80 percent – more than the 43 percent that would be expected if the effect were additive (Fig. 4A,B). The synergistic effect produced by co-injection of sub-threshold levels Xhepatocystin and XTRPM7 MOs suggested that Xhepatocystin and TRPM7 function in the same pathway. Thus, we next asked whether expression of TRPM7 could rescue the gastrulation phenotype caused by the Xhepatocystin MO. Surprisingly, given the number of N-glycosylated proteins, such as Frizzled receptors, that function to control gastrulation and neural fold closure, co-injection of mTRPM7 RNA with the Xhepatocystin MO was able to fully rescue the embryonic defect with a similar efficiency as co-injection of *murine* hepatocystin mRNA with the Xhepatocystin MO (Fig. 3D, lane 3–5)^21^. These results indicate that the gastrulation and neural fold closure defects caused by depletion of hepatocystin was primarily driven by disruption of TRPM7 function, but the mechanism involved still remained unclear.

### Hepatocystin augments TRPM7 protein expression

Glucosidase II is a soluble ER-resident heterodimer composed of two subunits^6^,^8^. Glucosidase II’s α subunit (GIIα) contains the glycosyl hydrolase active site, whereas Glucosidase II’s β subunit (hepatocystin) is required to sustain efficient N-glycan trimming by the interaction of its MRH domain and mannoses in the glycans^13^. The glycan structure in glycoproteins determines whether a glycoprotein is retained in the ER, sorted to its final normal destination or retrotranslocated to the cytosol to be degraded by proteasomes^10^,^11^,^12^,^22^,^23^. Thus, hepatocystin plays a determinative role in the quality control of glycoprotein folding in the ER. For example, loss of hepatocystin function impaired glucose trimming of PKD2, which likely accounts for its decreased expression in *Prkcsh*^*−/−*^ mice and the genetic interaction observed between *PKD2* and *PRKCSH* in ADPLD^17^,^18^.

**Figure 5:**
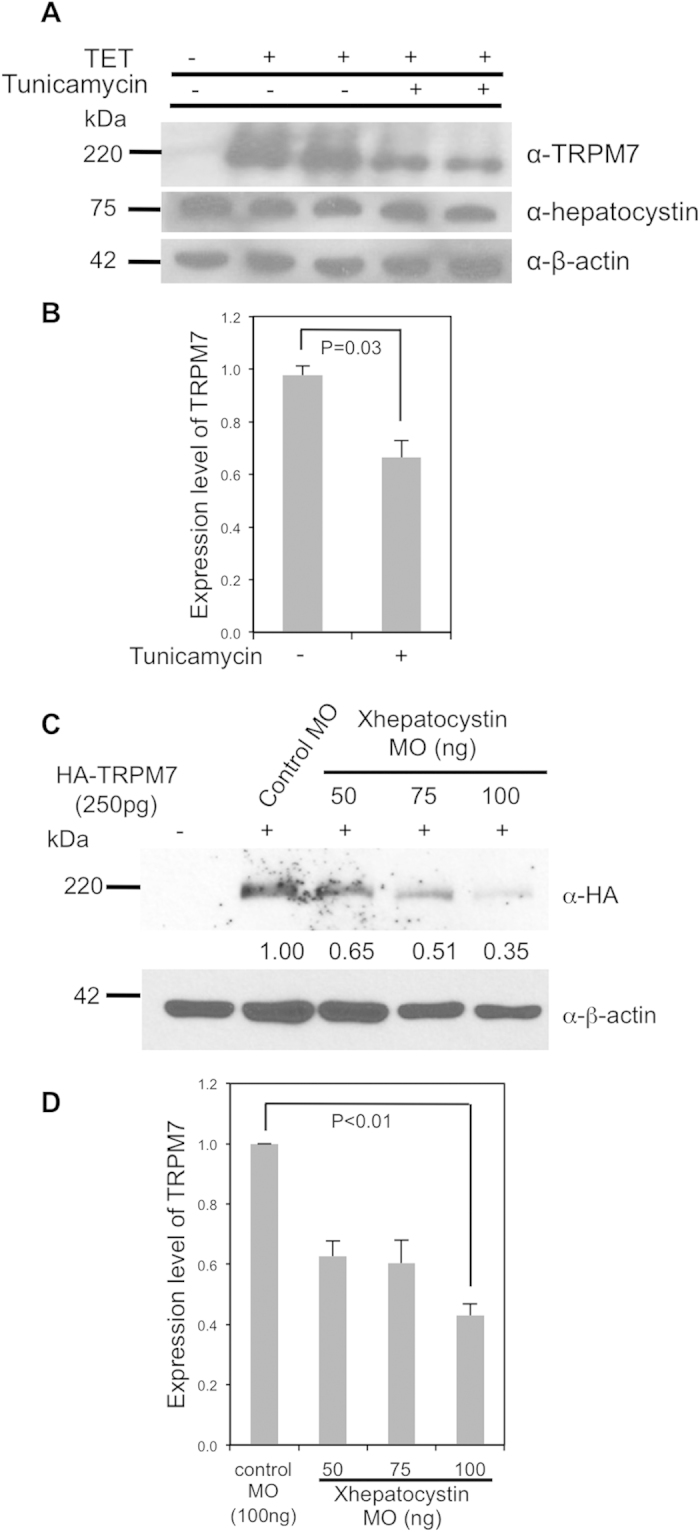
Hepatocystin and N-glycosylation affect the abundance of TRPM7. (**A**) Treatment of cells with tunicamycin (5 μg/ml) for 24 hours decreased tetracycline-induced TRPM7 protein expression in 293-TRPM7 cells. (**B**) Quantification of data from (**A**). (**C**) Co-injection of increasing dosages of the Xhepatocystin MO with 250 pg mRNA of HA-tagged mTRPM7 (HA-TRPM7) decreases TRPM7 expression in Xenopus embryos at stage 17. β-actin is shown as a loading control. (**D**) Quantification of data from (**C**). The experiments were repeated at least three times.

TRPM7 is reported to be N-glycosylated^24^. Given hepatocystin role in biogenesis of N-glycosylated proteins, we hypothesized that cellular depletion of hepatocystin could negatively impact TRPM7 protein expression. To examine the role of N-glycosylation in controlling TRPM7 expression and activity, we applied 5 μg/ml of tunicamycin to HEK-293 cells expressing TRPM7. The antibiotic tunicamycin selectively inhibits N-glycoslyation of membrane proteins and has previously been reported to interfere with TRPM7 N-glycosylation^24^,^25^. Tunicamycin treatment of 293-TRPM7 cells, transiently expressing TRPM7 in response to tetracycline, reduced channel expression (Fig. 5A,B), indicating that TRPM7 N-glycoslyation is important for the channel’s stability. We next investigated whether depletion of Xhepatocystin affects TRPM7 expression in *Xenopus laevis* embryos. Co-injection of Xhepatocystin MO with TRPM7 mRNA reduced TRPM7 protein expression in a dose dependent manner (Fig. 5C,D). Collectively, these results indicate a supportive role for Xhepatocystin in promoting TRPM7 protein expression and function during *Xenopus laevis* embryogenesis.

## Discussion

Our studies have uncovered a critical role for hepatocystin in supporting the function of the TRPM7 ion channel during embryogenesis. Like TRPM7, Xhepatocystin is ubiquitously expressed maternally to the tadpole stage and displayed a similar expression pattern as TRPM7 throughout development. In addition, depletion of Xhepatostatin in *Xenopus laevis* also produced gastrulation and neural fold closure defects, which offers explanation for the embryonic lethality of homozygous deletion of *PRKCSH* from mice. These results are also consistent with studies of *PRKCSH* in Zebrafish, where depletion of PRKCSH causes dorsal-flexure of the embryo, indicative of convergent extension defects, and situs inversus and pronephric cysts later in development^26^. The overlapping expression patterns of Xhepatocystin and XTRPM7 indicated the potential for these two proteins to functionally interact. Indeed, depletion of either Xhepatocystin or XTRPM7 interfered with gastrulation and neural fold closure and gastrulation phenotype produced by depletion of Xhepatocystin could be rescued by co-injection of mTRPM7 RNA. In addition, simultaneous depletion of XTRPM7 and Xhepatocystin by co-injection of subthreshold levels of their respective morpholinos produced a synergistic effect on the gastrulation phenotype, indicating that *PRKCSH* and *TRPM7* are epistatic and that the function of TRPM7 is dependent upon that of hepatocystin.

Depletion of Xhepatocystin in *Xenopus* embryos interfered with TRPM7 protein expression. Based on what is known regarding hepatocystin’s established function as the β-subunit of Glucosidase II, N-glycosylation and glycan trimming through Glucosidase II likely regulates the abundance of TRPM7 by limiting the channel’s turnover. Indeed, treatment of HEK-293 cells with tunicamycin decreased TRPM7 expression. Together these results provide a basis for further studies addressing the role of glycosylation in TRPM7 function.

Mutations in *PRKCSH* lead to Autosomal Dominant Polycystic Liver Disease (ADPLD), a rare hereditary disorder characterized by slowly progressive cyst formations. In addition to defects in *PRKCSH*, mutations in *SEC63* can also produce ADPLD^27^,^28^. SEC63 is an ER protein involved in mediating the translocation of nascent or newly synthesized polypetides across the ER membrane of cells. Thus both proteins implicated in ADPLD are involved in translocation through the ER membrane and in the oligosaccharide processing of newly synthesized glycoproteins^16^. Interestingly, only a few hepatic cysts are detectable in affected individuals in the first four decades of life, but hundreds of individual cysts can be found at a later stage^16^. It has been hypothesized that ADPLD cysts arise as a result of a cellular recessive two-hit mechanisms^16^. In the case of a germline *PRKCSH* mutation in ADPLD, the second hit could involve a somatic *PRKCSH* or *SEC63* mutation on the other allele, or somatic hits on genes implicated in Autosomal Dominant Polycystic Kidney Disease (ADPKD), such as *PKD1* and *PKD2*^16^. Mutations in *PRKCSH* or *SEC63* only account for 16–22% of cases of ADPLD, indicating that other genes contribute to this disease^29^.

Deletion of TRPM7 from mouse kidney leads to kidney cysts and a defect in nephrogenesis^30^. Whether, loss of TRPM7 function in liver contributes to the pathogenesis of ADPLD in response to *PRKCSH* deletion remains unknown. Recently, disruption of the Wnt pathway via mutations in *LRP5* was shown to be associated with hepatic cystogenesis^31^. In our previous work we demonstrated that TRPM7 functions within the non-canonical Wnt pathway during gastrulation and neural fold closure to regulate convergent extension cell movements^5^. This cellular process has also been shown to be required for tubulogenesis^32^. It has been speculated that malfunction of the non-canonical Wnt pathway could potentially result in tubule enlargement rather than tubule elongation^33^. Therefore, more studies are needed to assess the role of both the non-canonical Wnt pathway and TRPM7 in cystogenesis.

Around 30% of proteins are reported to undergo ER translocation and are subject to folding and quality control by hepatocystin and Sec63p^34^. This raises the question of the extent of the fidelity of hepatocystin’s interaction with TRPM7. TRPM7 is among only a select number of proteins, including TRPV4 and the inositol 1,4,5-trisphosphate receptor, that have been identified as hepatocystin binding partners via a yeast two-hybrid screen^23^,^35^,^36^. One possibility is that proteins that more specifically interact with hepatocystin may be subject to an additional layer of signalling control. Alternatively, binding of hepatocystin to TRPM7 may help target Glucosidase II activity towards the channel. Future studies will focus on how glycosylation affects TRPM7 function and how Glucosidase II is specifically impacting the channel.

## Methods

### Reagents

All cell culture reagents, unless otherwise stated, were from Life Technologies (Carlsbad, CA). All chemicals, unless otherwise stated were from Sigma Aldrich (St. Louis, MO). Carbenicillin, kanamycin, 5-Bromo-4-chloro-3-indolyl β-D-galactopyranoside (X-gal), isopropyl-beta-D-thiogalactopyranoside (IPTG) were purchased from Gold Biotechnology (St. Louis, MO).

### Plasmids and oligonucleotides

The pOG1 vector encoding human TRPM7 was previously described^37^. *Murine* hepatocystin in the pCMV-SPORT6 vector (Accession #: BC009816) was from GE Life Sciences (Piscataway, NJ). COOH-terminus FLAG-tagged hepatocystin was cloned into pcDNA5/FRT/TO (Life Technologies, Inc; Grand Island, NY) from the pCMV-SPORT6 vector using primers described in Supplementary Methods file.) To conduct pulldown purification assays we fused fragments of TRPM7′s COOH-terminus to a multifunctional Green Fluorescent Protein (mfGFP) in which GFP was engineered to contain a streptavidin binding peptide (SBP) tag, octa-histidine-tag, and c-Myc-tag in tandem into a loop of GFP^38^. mfGFP was subcloned into the NheI and HindIII sites of pcDNA6-V5-HisB to make pcDNA6-mfGFP. mfGFP-fusion proteins of TRPM7-COOH terminal fragments were subcloned by PCR into pcDNA6-mfGFP using pcDNA5/FRT/TO-HA-TRPM7 as a template^39^. The list of primers employed to make the mfGFP constructs are described in the Supplementary Methods file.

### Yeast two-hybrid (Y2H) screen and directed Y2H assay

The COOH-terminus of TRPM7 (residues 1120–1862) was subcloned into pBMT116 LexA vector to make the “bait” vector LexA-CTERM. A rat brain prey library in the pACT2 AD vector was purchased from Clontech Laboratories, Inc (Mountainview, CA). A yeast-two-hybrid screen was conducted following the manufacturer’s protocol for large-scale transformation. Positive interactions were identified by growth on selective media and a beta galactosidase assay before sequencing of purified plasmids. To conduct the directed Y2H beta galactosidase assay, the coiled-coil (CC) domain (residues 1120-1288) and kinase (KIN) domain (residues 1597–1862) of TRPM7 were subcloned into pBMT116 to make LexA-CC and LexA-KIN. The kinase-inactive LexA-CTERM (K1646R) mutant was made using QuikChange (Agilent Technologies, Santa Clara, CA) following the manufacturer’s protocol. The primers used to make the Y2H “bait” vectors are described in Supplementary Methods.

### Immunofluorescence and Confocal Microscopy

For immunocytochemical analysis of TRPM7 and hepatocystin, HEK-293T cells were plated onto coverslips and transfected with the pcDNA5/FRT/TO-HA-TRPM7 and pcDNA6-FLAG-hepatocystin plasmids. Cells were fixed at room temperature for 10 minutes in phosphate buffered saline (PBS) at pH 7.4 with 4% paraformaldehyde (Electron Microscopy Sciences; Hatfield, PA), and permeabilized with PBS containing 10% fetal bovine serum and 0.1% saponin. The rat monoclonal antibody Anti-HA antibody (clone 3F10, Roche Life Sciences; Indianapolis, IN) was used to visualize HA-tagged TRPM7 and mouse monoclonal Anti-FLAG M2 antibody (Sigma Aldrich; St. Louis, MO) was used to detect FLAG-tagged hepatocystin. Alexa Fluor 488 and Alexa Fluor 568 goat antibodies to rat and mouse (Life Technologies, Inc; Grand Island, NY) were used as secondary antibodies. Images were obtained at the Rutgers RWJMS CORE Confocal facility using a Yokogawa CSUX1-5000 microscope using 488-nm and 561-nm excitation wavelengths.

### Cell lines

The 293T cell line (CRL-3216) was purchased from American Type Culture Collection (Manassas, VA). The HEK-293 cell line expressing recombinant Hemaglutinin (HA)-tagged TRPM7 in tetracycline-inducible manner (293-TRPM7 cells) was previously described^21^. 293T and 293-M7 cells were maintained in a Dulbecco’s Modified Eagle Medium (DMEM), high glucose media with 10% FBS in a humidified 37 °C, 5% CO_2_ incubator

### Kinase Assay

To conduct the *in vitro* kinase assay, a GST-fusion protein containing TRPM7′s kinase domain (GST-KIN) was purified on a glutathione-agarose (Sigma Aldrich, St. Louis, MO) and dialyzed into 1X kinase buffer without ATP (20 mM Mops (pH 7.2), 100 mM NaCl, 20 mM MgCl_2_, 0.5 mM ATP, and 2 μCi of [γ-^32^P]ATP). A His-patch (HP) thioredoxin-fusion protein (Thio-hepatocystin) of hepatocystin (residues 346–528) was expressed in the pET102/D-TOPO vector (Life Technologies, Inc) and purified using HA agarose (Qiagen; Valencia, CA). HP thioredoxin-hepatocystin was dialyzed into 1X KIN Buffer and then subjected to an *in vitro* kinase assay using myelin basic protein (MBP) as previously described^3^.

### Embryo manipulations

Embryo manipulations were performed as previously described^40^,^41^. Embryo injections were performed using *in vitro* transcribed RNAs or Morpholino oligonucleotides (MO). The *Xenopus laevis* hepatocystin morpholino (Xhepatocystin MO) was 5- GCAGCAGCGCAAGCAAAGCCTTCAT-3 and was synthesized by Gene-Tools (Philomath, OR). The XTRPM7 MO2 was previously described^5^.

### Detection of protein expression

To detect HA-TRPM7 and actin expression in *Xenopus laevis*, embryos were lysed with lysis buffer (50 mM TRIS (pH 7.4), 150 mM NaCl, and 1% Igepal 630) and the proteins resolved by SDS-PAGE and western blotting using standard protocols. For detection of proteins in cultured cells lines, cells were lysed in the lysis buffer described above. The rat monoclonal antibody Anti-HA antibody (clone 3F10, Roche Life Sciences; Indianapolis, IN) was used to detect HA-tagged TRPM7. Anti-β-actin (sc-47778; Santa Cruz Biotechnology, Inc, Dallas, TX) was used to detect β-actin as loading control. Anti-GFP (sc-9996; Santa Cruz Biotechnology, Inc, Dallas, TX) was used to detect mf-GFP in pulldown purification assays. The anti-hepatocystin antibodies (catalogue # sc-374453, sc-10774; Santa Cruz Biotechnology, Inc. Dallas, TX) were used to detect endogenous hepatocystin in HEK-293 cells. HRP-linked secondary antibodies were goat anti-rat (sc-2006) and goat-anti rabbit (sc-2004). The sheep anti-mouse secondary antibody was purchased from GE Life Sciences (Piscataway, NJ). Transient transfection of cDNAs was conducted using TurboFect Transfection Reagent (Life Technologies, Inc; Grand Island, NY). For the co-immunoprecipitation experiment, 293-TRPM7 cells were treated with tetracycline (TET) at 1 μg/ml for 24 hrs to induce expression of HA-TRPM7. Following cell lysis, HA-TRPM7 was immunoprecipitated with monoclonal anti-HA-agarose (Sigma Aldrich, St. Louis, MO) overnight and the proteins resolved by SDS-PAGE and western blotting. For the pulldown purification assays using GST-hepatocystin, cDNAs for mfGFP-tagged TRPM7 fragments were transiently transfected into 293T cells and the proteins expressed for 24 hrs. Cells were lysed with lysis buffer and subjected to a GST-pulldown purification assay and the proteins resolved by SDS page and western blotting. For the tunicamycin experiment, 2 × 10^6^ TRPM7 cells were plated onto polylysine coated 60 mm dishes. The following day, tunicamycin (5 μg/ml) and tetracycline (5 μg/ml) were added. The cells were harvested 24 hours later and the cell lysate was subjected to SDS-PAGE and western blotting.

## Additional Information

**How to cite this article**: Overton, J. D. *et al.* Hepatocystin is Essential for TRPM7 Function During Early Embryogenesis. *Sci. Rep.* **5**, 18395; doi: 10.1038/srep18395 (2015).

## Supplementary Material

Supplementary Information
