# Supplementary material for: Hepatocystin is Essential for TRPM7 Function During Early Embryogenesis
Source: Sci Rep. 2015 Dec 16;5:18395. doi: 10.1038/srep18395 (PMC4680877; doi:10.1038/srep18395)
Supplement: Supplementary Information [file srep18395-s1.pdf]

Hepatocystin is Essential for TRPM7 Function During Early Embryogenesis

Jeffrey D. Overton[+,1], Yuko Komiya[+,1], Courtney Mezzacappa[2], Kaushik Nama[2], Na Cai[1], Liping Lou[1], Sorin V. Fedeles[3], Raymond Habas[2] & Loren W. Runnels[1,*].

[1]Rutgers-Robert Wood Johnson Medical School, Dept. of Pharmacology, Piscataway, 08854, U.S.A.
[2]Temple University, Dept. of Biology, Philadelphia, 19122, U.S.A.
[3]Yale University School of Medicine, Dept. of Internal Medicine, New Haven, 06510. USA.
*runnellw@rwjms.rutgers.edu
[+]These authors contributed equally to this work.

Supplemental Information

Protein	# of Hits
hepatocystin (80K-H; Glucosidase Iiβ)	4
BLOS1 (GCN5L1/RT14)	6
BLOS2	1
Dysbindin	3
Proteasome 26S non-ATPase subunit 12	1
Proteasome 26S non-ATPase subunit 1	1
Hematopoietic PBX-interacting protein	1
CDC5-like protein	1
KRMP1 like protein	1
Smarce1R	1
Prosaposin	2
KIAA0092	1
Bile acid receptor	2
C11ORFF13	1
pVHL-interacting deubiquitinating enzyme	1
BAT3	1
JTV1	1
Clusterin	1
DNA methyltransferase associated protein	1
MIP-T3	1
14-3-3θ	1

Supplementary Table 1. Positives From Yeast Two Hybrid Screen. Yeast two hybrid screen of a mouse brain prey library using the COOH-terminal domain of TRPM7 as “bait” identified 20 different proteins with a combined 32 “hits”.

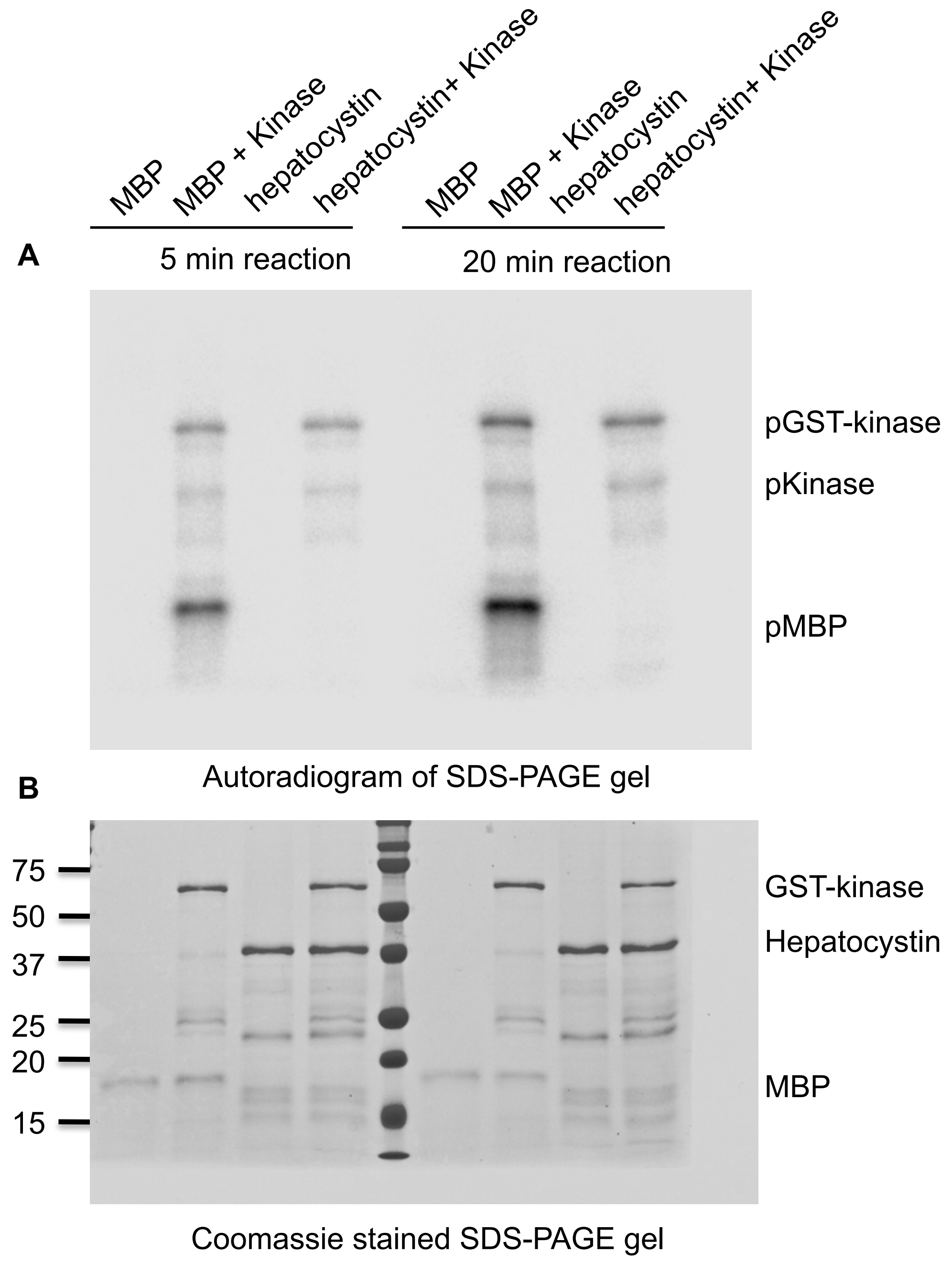

Supplementary Figure 1. TRPM7 does not phosphorylate hepatocystin.
(A) Autoradiogram of SDS-PAGE gel after electrophoresis of phosphorylation reactions containing GST-kinase fusion proteins with or without myelin basic protein (MBP) and hepatocystin. (B) Coomassie-stained gel.

Supplementary Methods

Cloning of multifunctional-GFP TRPM7 fusion proteins
Multifunctional GFP fusion proteins of TRPM7 fragments were made in the pcDNA6-mfGFP vector by PCR based cloning from pcDNA5/FRT/TO-TRPM7 using the following primers:

GFP-CTERM 5'-CAT CAT GGT ACC ATG GCT TAT CAT GAA AAA CCA GTC CTG CCT C-3' (forward primer/KpnI).

GFP-CTERMΔCC 5'- CAT CAT GGT ACC ATG AAA GAA ACC TAG TGC TGT AAA CAC A-3' (forward primer/KpnI).

GFP-CC forward primer was the same as GFP-CTERM.

GFP-ST forward primer was the same as GFP-CTERMΔCC.

GFP-CC forward primer was the same as GFP-1288.

GFP-KIN 5'-CAT CAT GGT ACC AGC ATG TCT TCA TGG TCT CAG CTA GGC- 3' (forward primer/KpnI).

The reverse primer for GFP-CTERM and GFP-CTERMΔCC was:
5'- ATG ATG GGA TCC CTA TCC CTA TAA CAT CAG ACG AAC AGA ATT TGT TGC-3' (reverse primer/BamHI).

The reverse primer for GFP-CC was:
5'- ATG ATG GGA TCC CTA CAA AGG TCT TAC AGG AAC ATC ATC AAT AAG ATT-3' (reverse primer/BamHI)

The reverse primer for GFP-KIN was the same as GFP-CTERM

GFP-ST reverse primer
5'-ATG ATG GGA TCC CTA ATT CAG TAT ACT GGG AGA ACT CTC CTC CAG-3' (reverse primer/BamHI).

Cloning of yeast two-hybrid (Y2H) TRPM7 bait vectors
LexA fusion proteins of TRPM7 fragments were made in the pBMT116 LexA bait vector by PCR based cloning from pcDNA5/FRT/TO-TRPM7 using the following primers:

LexA-CTERM FORWARD
5'-CAT CAT GGA TCC GTA TGG CTT ATC ATG AAA AAC CAG TCC TGC-3'

LexA-CC
(LexA-CC) forward primer was the same as LexA-CTERM FORWARD

LexA-CTERM REVERSE
5'- ATG ATG GGA TCC CTA CAA AGG TCT TAC AGG AAC ATC ATC AAT AAG ATT-3'

LexA-KIN FOR

5'-CAT CAT GGT ACC GTA GCA TGT CTT CAT GGT CTC AGC TAG GC- 3' (forward primer/KpnI).

The reverse primer for LexA-KIN was the same as LexA-CTERM.

Primers used for QuikChange to introduce the K1646R mutation was:

5'-CCT GAA GTC AGG GCA TCT CTA TAT CAT TCG GTC ATT TCT TCC TGA GGT G-3'

5'-CAC CTC AGG AAG AAA TGA CCG AAT GAT ATA GAG ATG CCC TGA CTT CAG G-3'

Correct orientation and verification that the fragment was in-frame was verified by sequencing with pBMT166 forward primer
5'-CGA ACT GTT GCC AGA AAA TAG CGAG -3'

Cloning of COOH-terminus FLAG-tagged hepatocystin

5'–CAT CAT GGT ACC ATG CTG CTG CTG CTG CTA CTA CTA CTA C-3' (forward primer/kpnI)

5'-ATG ATG GGA TCC CTA CTT GTC ATC CTT GTA ATC CAG CTC GTC ATG GTC CCC ATC-3' (reverse primer, BamHI)
